# Retraction: Epithelial Mesenchymal Transition Is Required for Acquisition of Anoikis Resistance and Metastatic Potential in Adenoid Cystic Carcinoma

**DOI:** 10.1371/journal.pone.0108117

**Published:** 2014-09-10

**Authors:** Jun Jia, Wei Zhan, Jian-Ying Liu, Gang Chen, Hui Liu, Hao-Yan Zhong, Bing Liu, Yu Cai, Jia-Li Zhang, Yi-Fang Zhao

The authors retract this publication due to concerns about the cell lines employed in the study.

The study reports experiments looking at pathways involved in anoikis resistance in human adenoid cystic carcinoma; the experiments involved the use of the cell lines ACCM and ACC2. After the publication of the article, a reader raised concerns that these two cell lines may not originate from adenoid cystic carcinoma. The authors have completed a short tandem repeat analysis on the cell lines and this has revealed cross-contamination with other human cells, which compromises the relevance of the work to human adenoid cystic carcinoma, and thus the conclusions of the study.

In the light of this, the authors retract this publication.
